# Analysis of the Effect of an Open Hole on the Buckling of a Compressed Composite Plate

**DOI:** 10.3390/ma17051081

**Published:** 2024-02-27

**Authors:** Pawel Wysmulski

**Affiliations:** Faculty of Mechanical Engineering, Department of Machine Design and Mechatronics, Lublin University of Technology, Nadbystrzycka 36, 20-618 Lublin, Poland; p.wysmulski@pollub.pl

**Keywords:** buckling, critical load, post-buckling, FEM

## Abstract

This paper investigates the effect of an open hole on the stability of a compressed laminated composite plate. The study was carried out in two ways: using experimental tests and numerical analysis. As a result of the experiment, the buckling form and path of the plate were recorded. The form of buckling was determined using the ARAMIS non-contact measurement system. The critical load value was determined from the working path using the approximation method. The experimental results were verified by numerical analysis based on the finite element method. FEM investigations were carried out in terms of a linear eigenproblem analysis. This allowed the bifurcation load and the corresponding buckling form of the numerical model of the plate to be determined. Investigating the effect of the hole in the compressed plate at a critical state showed high agreement between the proposed test methods. No clear effect of the hole size on the buckling of the plate was observed. In contrast, a clear effect of the hole on the critical load value was determined. The maximum decrease in the critical load value was 14%. The same decrease was observed for the stiffness of the post-critical characteristics. It was shown that the [45|−45|90|0]s composite plate had more than three times lower strength compared to [0|−45|45|90]s and [0|90|0|90]s. The novelty of this article is the development of a research methodology based on new interdisciplinary research methods for describing the influence of the central hole on the stability of compressed composite plates. The ABAQUS system was used for the numerical analysis.

## 1. Introduction

Thin-walled structures belong to a group of load-bearing structures characterised by significantly higher strength and stiffness in relation to their own weight. This determines the wide use of this type of structure in the aerospace [1,2] and automotive industries [3,4] as well as in other areas of modern engineering. Modern design of thin-walled load-bearing structures is seeing an increase in the use of modern structural materials such as laminate composites in place of traditional isotropic materials. The popular widely used category of composite materials are polymer composites reinforced with glass, carbon and Kevlar fibres. These materials have a significant effect on increasing the strength properties of thin-walled structural components.

An important issue in the design of thin-walled load-bearing structures is the possibility of the occurrence of loss of stability and load-carrying capacity by the elements of the structure and, in particular, the knowledge of the critical load and stiffness of the structure in post-critical states [5,6,7]. This knowledge has become important and necessary because there are papers in which the critical load corresponding to the buckling of the structure is regarded as the limit load [8,9]. Many researchers have addressed the issue of local elastic buckling of composite structures in their studies [10,11]. One may also find papers supported by experimental results on the buckling of composite plate structures [12,13,14]. The static character of such structures allows the ability to carry the load further in the post-buckling state. For this reason, it becomes reasonable to extend the research to include a post-critical state, describing the non-linear stability issue of thin-walled structures [15,16,17,18]. In the papers [19,20,21], the limit states of compressed composite structures up to the complete loss of their load-bearing capacity were described.

Determination of the stress concentration factor (SCF) around the notch is also highly required for the design of thin-walled structures. There are many studies on stress concentration in the structural behavior of coatings with different cut-outs [22,23,24]. In addition, with the advancement of computational tools and considering the difficulty of conducting experimental and statistical studies, they have become the preferred tools for evaluating the state of SCF in mechanical structures [25,26] and have also been recognized as an effective tool in the finite element method [27,28,29,30]. In this context, it is necessary to better understand the main parameter affecting SCF in thin cylindrical shells subjected to axial compression [31] as well as uniform lateral pressure [32,33,34].

The need for a hole in thin-walled elements is unavoidable for design reasons. The use of a hole weakens the structure and affects its strength [35,36,37,38]. This has been described in many articles in the context of isotropic materials. However, there is a lack of work devoted to examining the impact of the hole on the strength of structures made of layered composites. The occurrence of a hole in layered composites becomes a complex issue because, in laminates, the material properties change from layer to layer. For this reason, the study explored the issue of the influence of an open hole on the buckling of a compressed composite plate.

The manuscript investigated the effect of the size of the central circular open hole on the stability of a thin-walled plate element made of laminate. The study included linear and non-linear numerical analysis of the structure using the finite element method (FEM) and experimental verification. The novelty of the study is the use of interdisciplinary research methods to investigate the effect of the central hole on the stability of compressed plates made of a layered composite. Numerical analysis based on the finite element method is nowadays becoming an increasingly accepted tool used to investigate current scientific issues [39,40,41,42,43,44,45].

## 2. The Subject of Study

The plates chosen for the study were made from a carbon fiber-reinforced laminate composite [46,47,48,49,50]. The material was an eight-ply laminate in a symmetrical fiber layout with respect to the center plane of the plate. The composite plates were characterized by three standard fiber layouts, as presented in Table 1.

**Figure 1:**
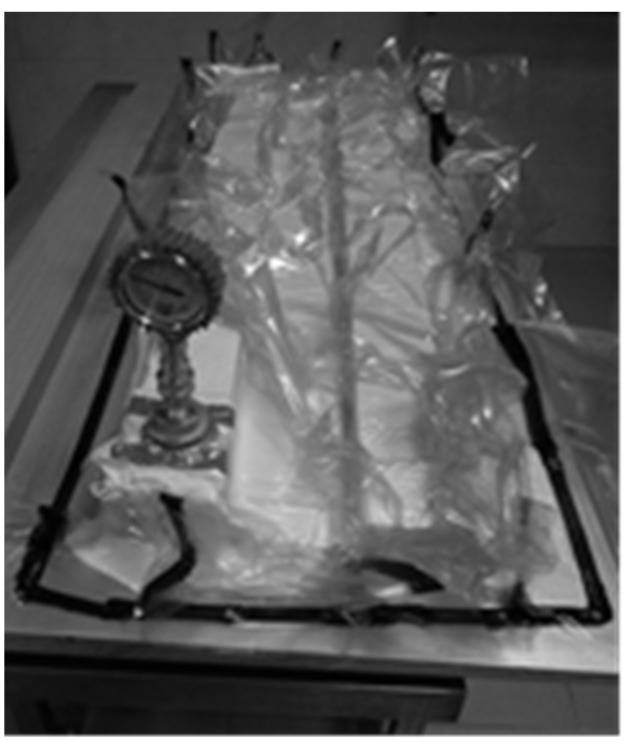
Process of manufacturing composite samples—vacuum package.

The plates were made prepregs using the popular autoclave method. Manufacturing using this method takes place in a device called an autoclave at a time-controlled temperature and pressure, and the action of negative pressure on the product enclosed in a vacuum bag—Figure 1.

**Figure 2:**
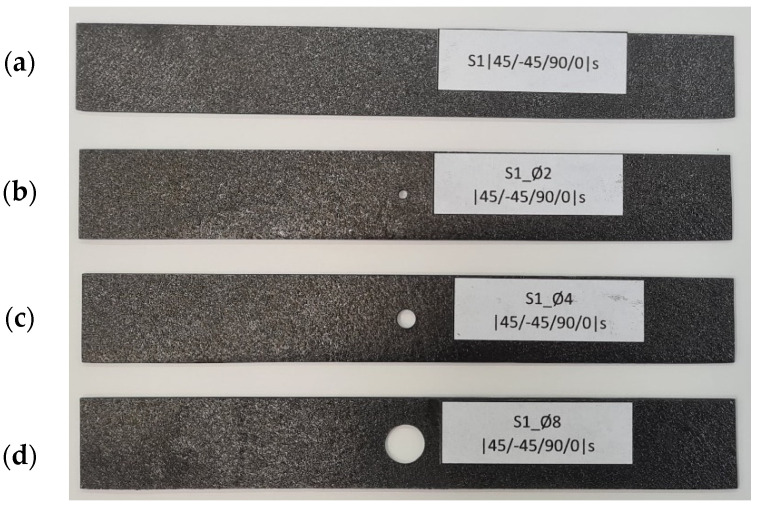
Real CFRP plates: (**a**) plate without a hole, (**b**) plate weakened with a Ø2 mm hole, (**c**) plate with a Ø4 mm hole, (**d**) plate with an Ø8 mm hole.

The material produced using this method was characterized by low porosity, uniform fiber placement, and high repeatability of manufacture. Samples produced using this method are presented in Figure 2.

The plates were characterized by dimensions: width 20 mm, length 140 mm, and thickness 1.048 mm (single layer thickness 0.131 mm). Selected sizes of through-holes were made at the center points of the plates. It was decided to make three plates with a different hole for each composite configuration. The specimens were drilled with two times the size of the holes, resulting in specimens with through-hole diameters of 2 mm, 4 mm, and 8 mm—Figure 2. The quality of the holes in the composite plates was within engineering tolerances.

**Figure 3:**
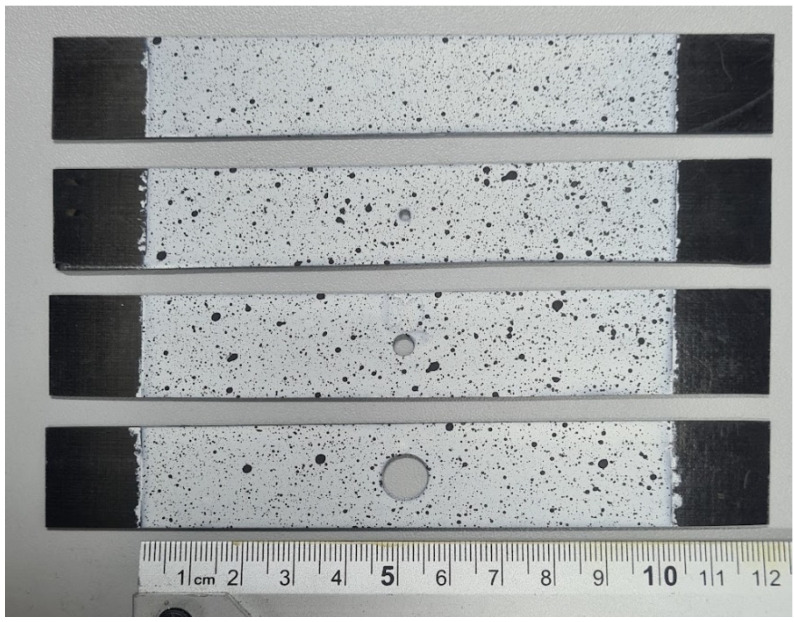
Plates painted with contrasting patterns.

The test samples were also painted with a contrast pattern in order to observe their deformation with the non-contact measuring system, ARAMIS. For this, it was necessary to determine the measuring field, which was 20 × 100 mm, and to apply white paint and then graphite contrast cents. The samples prepared in this way are presented in Figure 3.

The material properties of the CFRP composite used were determined by experimental tests in accordance with the ISO standard. The mechanical and limiting properties of the laminate used are presented in Table 2.

## 3. Methodology

The analysis focused on an axially compressed composite plate with an open circular hole of selected diameter in the critical state (linear problem) and in the post-critical state (non-linear stability problem). The scope of the study was twofold: experimentally on the real plate and numerical analysis using the finite element method (FEM). Experimental testing of the composite plate made it possible to observe the real behavior of the structure in the critical and post-critical states. The non-contact ARAMIS system was used to identify the buckling form, while the corresponding critical load value was determined using an approximation method. The independent numerical simulation carried out consisted of modeling appropriate FE models. The buckling of the model was obtained by solving the linear eigenproblem, while the solution of the non-linear stability problem was based on computations carried out on models with initiated geometrical imperfection corresponding to the lowest form of buckling of the structure. The analysis carried out allowed the buckling of the real plate to be represented correctly.

## 4. Experimental Study

**Figure 4:**
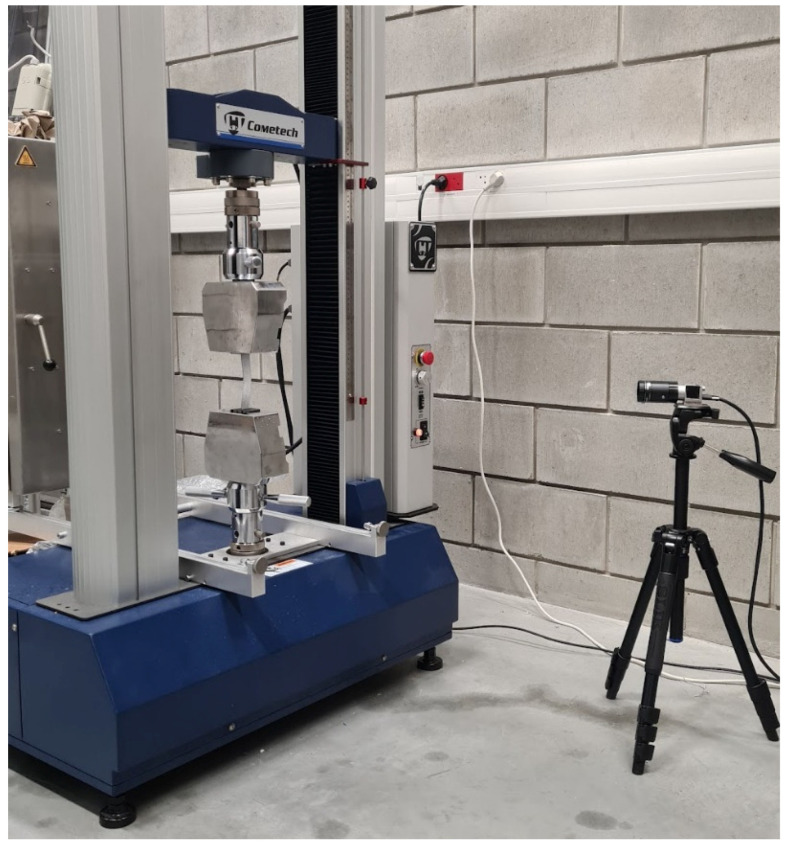
Test stand.

Experimental testing of the compression hole composite plate was carried out using a Cometech universal testing machine equipped with a 2.5 kN load cell at a constant crosshead displacement speed of 1 mm/min. The prepared plate was clamped in the machine’s grips to create a 20 × 100 mm test area with a centrally located open hole. The ends of the plate were pushed into the grips to a dimension of 20 mm (unpainted areas of the test specimen—Figure 3). Experimental testing enabled the compressive load and shortening of the specimen to be recorded over the time period of the measurement. The deformation process of the specimen during the measurement was recorded using the ARAMIS non-contact optical measurement system [52,53]. The result of the experiment was the determination of the structure’s working characteristics, which made it possible to describe the critical and post-critical state of the compressed hole plate. The view of the complete test stand with the restrained specimen and measurement system is presented in Figure 4.

Experimental investigations are subject to various types of inaccuracy, which could affect the quality of the results. These include geometrical imperfections in the test specimen and non-ideal boundary conditions. In such cases, the experimental working characteristics of the plate are subject to defects from the outset, the compression being accompanied by an additional bending state. On the basis of such characteristics, it has been difficult to clearly determine the value of the critical load, as they do not have a bifurcation point (the point at which the characteristics change from linear to non-linear). In such cases, approximation methods are used to determine the local critical load on the basis of the measurements obtained in experimental tests. In this study, the popular straight-line intersection method was used to assess the critical load value. The straight-line intersection method consisted of approximating the post-critical work path, which is the ratio of the sample load and shortening. The post-critical working path *P*-*u* was approximated in selected intervals by two linear functions. The value of the critical load describes the point of intersection of the determined approximation lines. It is determined by solving the following system of equations [54,55]:

(1) $$\{\begin{array}{c} P_{cr}=P\frac{a_{1}}{a_{0}}u+P\\ P_{cr}=P\frac{a_{2}}{a_{0}}u+P \end{array}\to \;\left(P_{cr};\;u\right)$$

- *a*_1_, *a*_2_—unknown parameters of the function,
- *P*—load,
- *P_cr_*—critical load,
- *u*—shortening of the plate corresponding to the critical load.

## 5. FEM Analysis

Numerical computations were carried out in two stages: linear and non-linear range. The first stage was an analysis of the critical state of the structure using stability analysis to determine the bifurcation load on the compressed plate model and the associated buckling mode [56]. The stability issue of such structures was determined by solving [57]:

(2) $$\left(\left[K\right]+\lambda_{i}\left[H\right]\right){\left\{\psi \right\}}_{i}=0$$

- [*K*]—structural stiffness matrix,
- [*H*]—stress stiffness matrix,
- *λ_i_*—*i*-th eigenvalue,
- *ψ*—*i*-th eigenvector of displacement.

The Equation (2) was satisfied when the eigenvector of the displacement was 0, or the determinant of the expression in parentheses was 0. If {*ψ*}*_i_* = 0, then this was a trivial solution. This meant that the structure remained in the initial equilibrium state. The bracketed expression in (2) gave the following solution:

(3) $$\left|\left[K\right]+\lambda_{i}\left[H\right]\right|=0$$

The Equation (3) presented an eigenvalue problem to find *n* values of the buckling load multiplier λ and the corresponding buckling mode shape.

**Figure 5:**
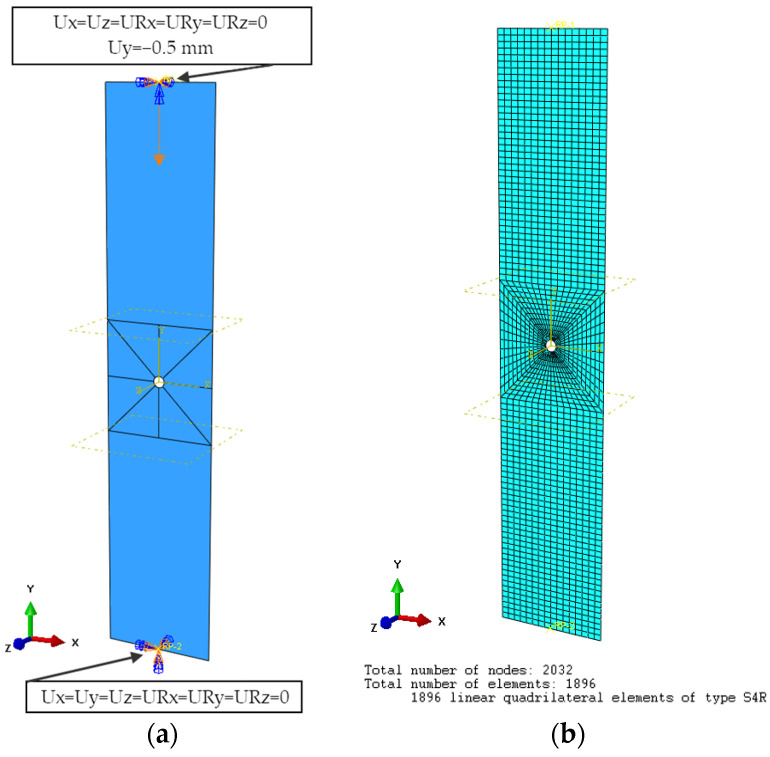
FEM model: (**a**) boundary conditions, (**b**) discrete plate model.

The second stage of the simulation was the solution of the non-linear stability problem, in which computations were carried out on a FEM model with implemented geometric imperfection corresponding to the first form of buckling of the plate. SHELL-type shell finite elements with six degrees of freedom at each node were used to discretize the model. The type of finite element used was a four-node shell element with reduced integration, designated S4R—Figure 5b. The process of discretizing the FEM model began with its partition. For this purpose, a 20 × 20 mm area was extracted in the middle of the plate and divided into eight equal partitions. This enabled the use of a high-quality finite element mesh (Structured). The structured meshing provided the most control over the mesh, as it applied predetermined mesh patterns to specific model topologies. The finite element size was adopted on the basis of a preliminary numerical analysis, which showed that reducing the finite element size did not affect the quality of the numerical results. The global finite element size of 1 × 1 mm was adopted with local compaction of the hole perimeter. This approach made it possible to similarly discretize plates with a 2 mm, 4 mm, and 8 mm hole. Decreasing the element size near the holes did not affect the results but significantly increased the computation time. The structure of the laminate was modeled using the LAYUP-PLY technique, with which the configuration of the composite layers was determined. The properties of the composite material were described by defining an orthotropic material model in the plane stress state. The mechanical properties of the material were assumed according to Table 2. The numerical model had dimensions of 20 × 100 × 1.048 mm and was designed to represent only the measurement area of the experiment. The boundary conditions corresponded to the realization of the experimental restraint of the end areas of the composite plate. As part of this, reference points were defined as located at the center of the top and bottom edges of the plate, which took away all six degrees of freedom from the edges. The lower reference point of the plate was blocked from all six degrees of freedom; the upper reference point was blocked from displacement with respect to the X and Z axes, and all rotational steps were blocked—Figure 5a. The compressive load on the model was the displacement response of the upper reference point Uy = −0.5 mm. Numerical computation based on the finite element method was performed in Abaqus.

## 6. Discussion of the Results

**Figure 6:**
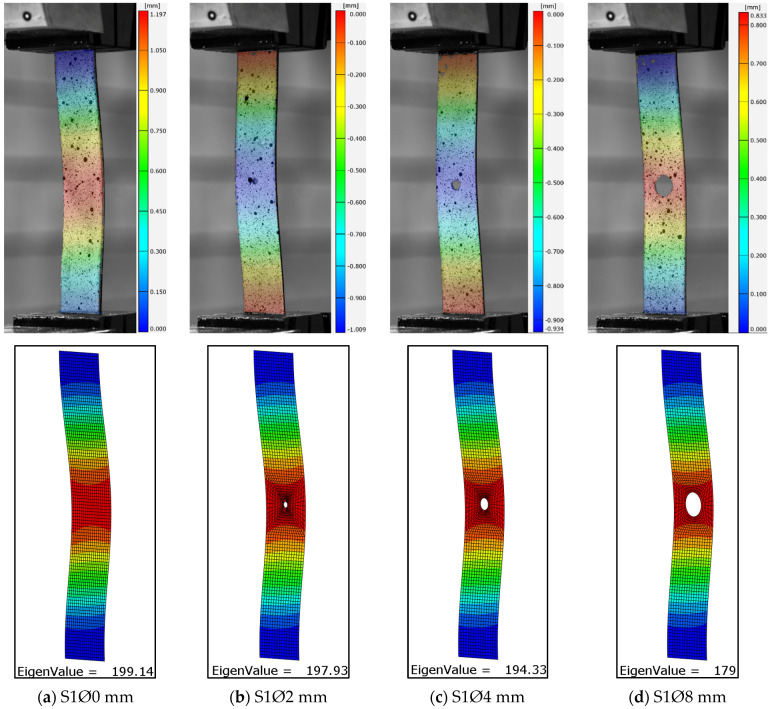
Lower experimental, numerical form of buckling of plates [45|−45|90|0]s: (**a**) plate without a hole, (**b**) plate with a Ø2 mm hole, (**c**) plate with a Ø4 mm hole, (**d**) plate with a Ø8 mm hole.

**Figure 7:**
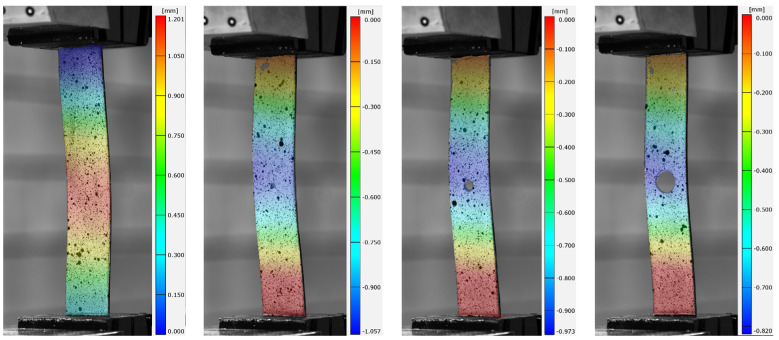

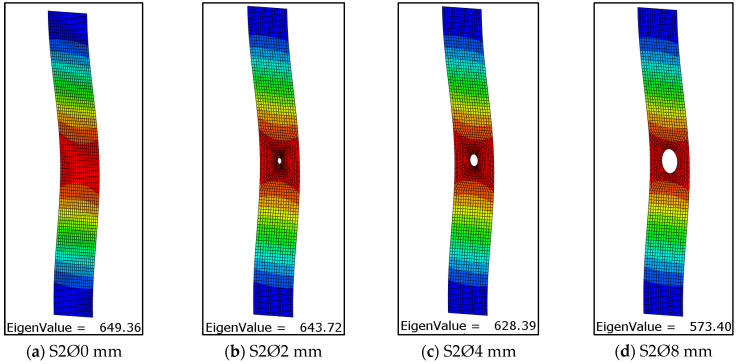
Lower experimental, numerical form of buckling of plates with a hole in [0|−45|45|90]s: (**a**) without a hole, (**b**) with a Ø2 mm hole, (**c**) with a Ø4 mm hole, (**d**) with a Ø8 mm hole.

**Figure 8:**
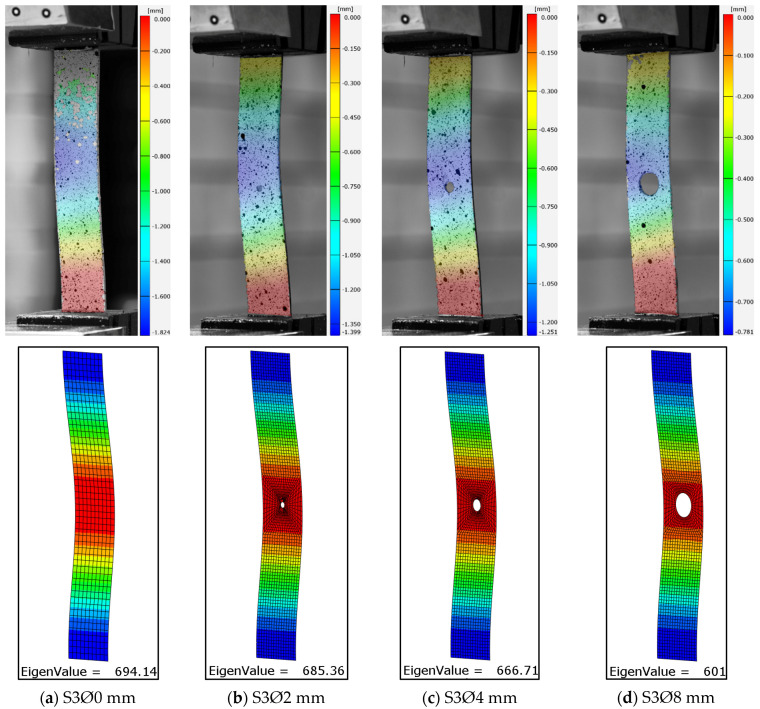
Lower experimental, numerical form of buckling of plates with a hole in the [0|90|0|90]s: (**a**) without a hole, (**b**) with a Ø2 mm hole, (**c**) with a Ø4 mm hole, (**d**) with a Ø8 mm hole.

Experimental investigations allowed the influence of the size of the open hole on the buckling of the compression composite plate to be described. The obtained test results enable a qualitative and quantitative analysis of the critical state based on the recorded test parameters. Identification of the critical state of the test item was carried out on the basis of the first buckling form obtained and the corresponding critical load value [58]. The experimentally determined critical value formed the basis for verifying the results of the numerical FEM calculations. The obtained experimental deformation and the lowest numerical form of buckling of the tested open-hole composite plates are presented in Figure 6, Figure 7 and Figure 8.

In order to obtain a more extensive representation of the experimental form of plate deformation, a mean displacement map was calculated on the basis of the ARAMIS optical measurement and applied to the real photographs. As assumed, the largest displacements were obtained in the area where the holes occurred. All analyzed cases were characterized by the formation of one local half-wave. The deformation forms obtained for all the plates studied corresponded to the first numerical forms of buckling. In all cases, the effect of hole diameter size did not change the buckling form of the plate. Similarly, changing the configuration of the composite layers did not change the form of buckling. Qualitative analysis of the results confirms the conformity of the numerically simulated buckling forms of the thin-walled plate with the form of deformation obtained in experimental tests (Figure 6, Figure 7 and Figure 8). The resulting numerical buckling form was used as the initial geometry for non-linear FEM analysis.

**Figure 9:**
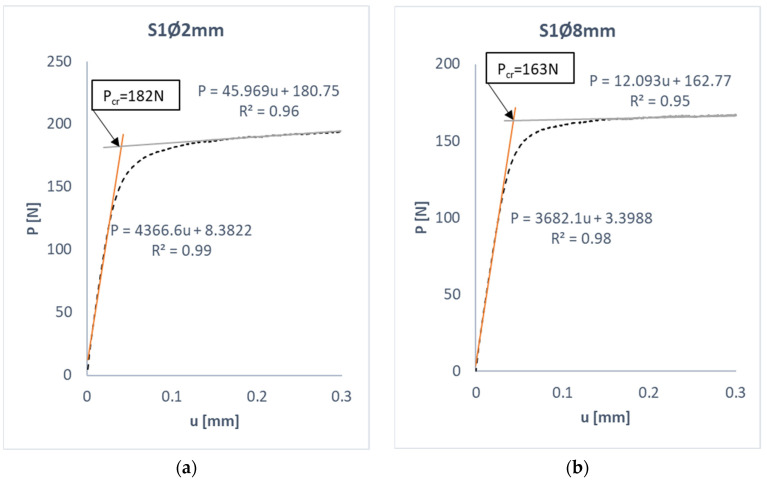

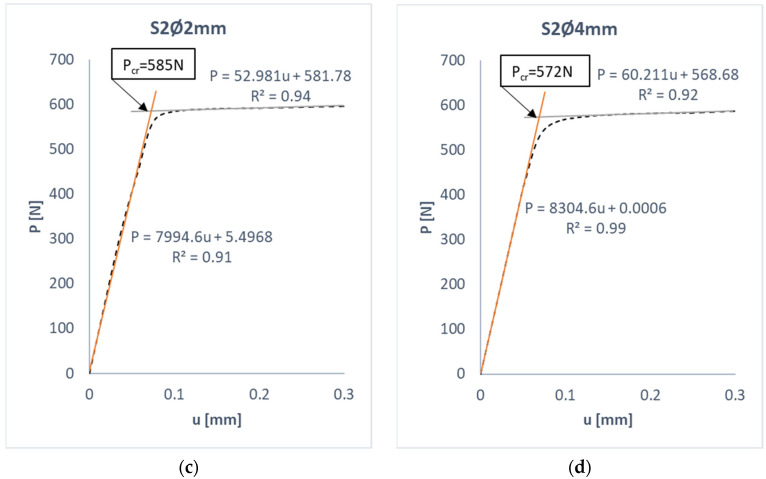
Determination of the critical load by the straight-line intersection method: (**a**) S1Ø2 mm, (**b**) S1Ø8 mm, (**c**) S2Ø2 mm, (**d**) S2Ø4 mm.

Quantitative analysis of the results obtained makes it possible to determine the value of the critical load corresponding to the lowest form of loss of stability plate studied. Based on the experimental measurements of shortening as a function of load, the mean values of the critical load were determined using the straight-line intersection approximation method. Figure 9 presents the application of this method to determine the approximate critical force from experimental tests.

Using the straight-line intersection method, the experimental critical load values were determined for all tested plates. In parallel, numerical simulation solved the eigenproblem, which resulted in a critical (bifurcation) load value corresponding to the lowest buckling mode. This made it possible to compare the experimental and numerical values of the critical load—Table 3. The results in the table were compared with each other, and the relative error calculated using the formula was determined:

(4) $$\delta =\frac{\left|FEM-EXP\right|}{FEM}\cdot 100\%$$

- *δ*—relative error,
- *FEM*—numerical results,
- *EXP*—experimental values.

**Table 3:** Critical load values.

			S_Ø0 mm	S_Ø2 mm	S_Ø4 mm	S_Ø8 mm
S1 [45|−45|90|0]s	FEM	[N]	199	198	194	179
	EXP	[N]	187	182	178	163
	relative error	[%]	6.03%	8.08%	8.25%	8.94%
S2 [0|−45|45|90]s	FEM	[N]	649	644	628	573
	EXP	[N]	610	585	572	524
	relative error	[%]	6.01%	9.02%	8.92%	8.55%
S3 [0|90|0|90]s	FEM	[N]	694	685	667	601
	EXP	[N]	639	628	619	553
	relative error	[%]	7.93%	8.32%	7.20%	7.99%

The results presented in Table 3 show a high quantitative agreement between the critical (bifurcation) load values of the numerical calculations and the experimental results. The value of the critical load determined by these methods had a relative error of no more than 9%, which, in the case of stability analysis of thin-walled structures, confirms the correctness of the test methods used. It should be added that the numerical calculations represent an ideal solution and should represent an upper estimate of the critical load, while the experimental results are subject to the inaccuracies of the experiment and could be represented as a lower estimate of the critical load.

**Figure 10:**
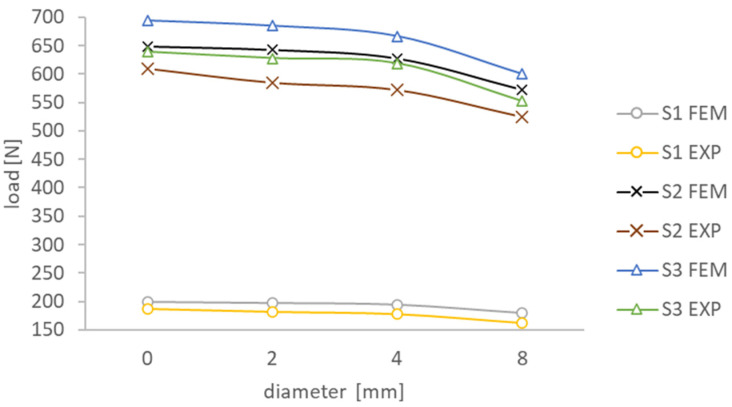
Effect of the hole on the critical load of the compressed plate.

The influence of the open hole size on the critical behavior of the compressed composite plate was assessed. For this purpose, a plot of the obtained critical loads with respect to the tested holes was developed, which is presented in Figure 10. For all tested plates, the hole size influenced a monotonic decrease in the critical load value. The largest decrease in the experimental value of the critical load occurred for plate S2 and was 14.1%, and the smallest for S1 was 12.8%. It should be added that the S1 plate with a composite configuration of [45|−45|90|0]s had more than three times lower strength compared to S2 [0|−45|45|90]s and S3 [0|90|0|90]s. This is evidenced by the displacement of the stiffest layer with the [0] configuration to the center of the layer stack (to the plane of symmetry), thus weakening the S1 plate.

**Figure 11:**
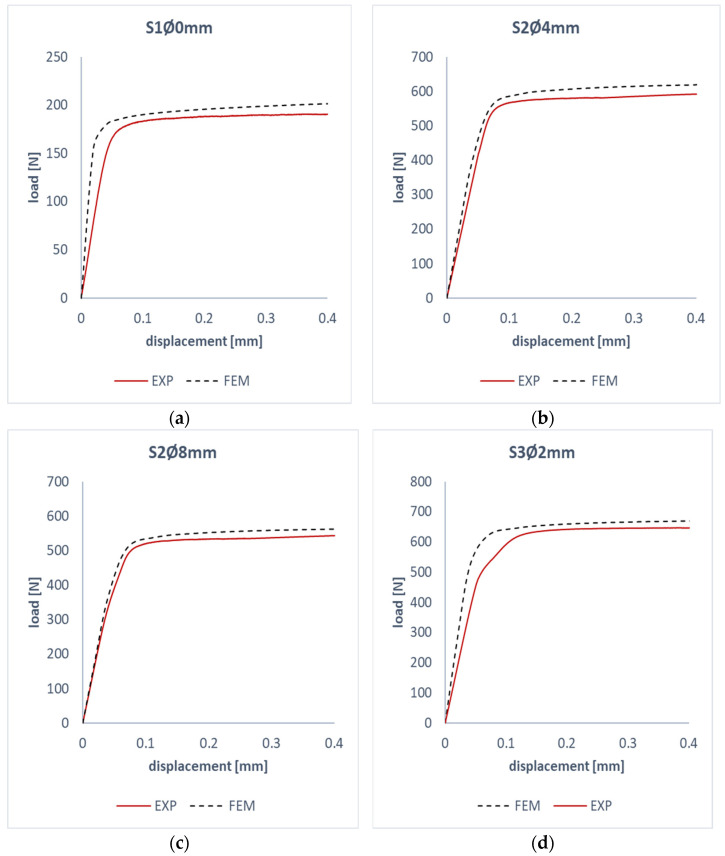
Comparison of experimental and numerical working paths: (**a**) S1Ø0 mm, (**b**) S2Ø4 mm, (**c**) S2Ø8 mm, (**d**) S3Ø2 mm.

The second stage of numerical analysis was to solve the problem of non-linear stability of the plate in the post-critical state using the lowest form of buckling. This allowed for the determination of post-critical paths based on the FEM calculations. Experimental testing continued in the range of up to 150% of the critical load. This made it possible to determine the characteristics of the tested plates in the post-critical state. Figure 11 shows a comparison of experimental–numerical post-critical work paths. The obtained characteristics show a similar path; this confirms the validity of using the selected test methods to investigate the non-linear state of the compressed plates. In all the tested examples, the assumed trend was obtained, and the numerical post-critical characteristics were stiffer than the experimental ones. This may be evidenced by the fact that the numerical analysis compared to the experiment represents ideal test conditions.

**Figure 12:**
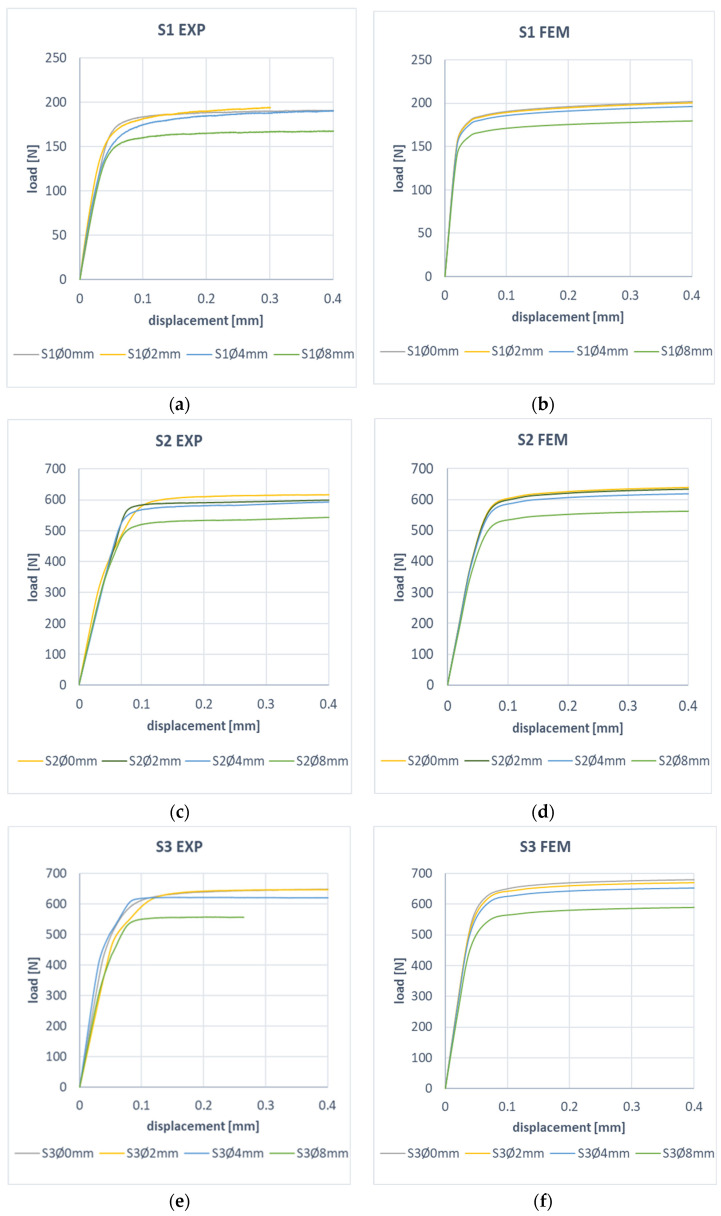
Effect of hole size on post-critical plate behavior: (**a**) S1 EXP, (**b**) S1 FEM, (**c**) S2 EXP, (**d**) S2 FEM, (**e**) S3 EXP, (**f**) S3 FEM.

In order to demonstrate the effect of the hole of the compressed composite plate in the post-critical state, the determined work paths were analyzed. The experimental and numerical characteristics of the post-critical characteristics for plates S1, S2, and S3 are presented in Figure 12. For all plates, a change in the stiffness of the post-critical characteristics was observed with an increase in the diameter of the hole in the plate. Experimental results show that the decrease in stiffness of the post-critical characteristic is for plate: S1 14% (FEM 11%), S2 12% (FEM 11%), and S3 10% (FEM 11%). Based on the high convergence of the presented results, the correctness of the proposed FEM model was confirmed, which could be used in this type of analysis.

## 7. Conclusions

The study investigated the effect of the size of the central open hole on the critical and post-critical behavior of a compressed composite plate. Two independent test methods were selected for the study. The developed numerical simulation based on FEM was successfully verified by experimental tests. In addition, the paper demonstrates the effect of the ply layout on the strength of the compressed plate with the hole.

- The study of the effect of the hole of the compressed plate in the critical state showed high agreement between the proposed test methods. No clear effect of the hole size on the form of buckling of the plate was observed. In all cases, one local half-wave was formed.
- On the other hand, a clear effect of the hole on the value of the critical load was determined. The maximum decrease in the value of the critical load equal to 14% was obtained for the plate with the S2 fiber layout, while the minimum decrease was 13% for the S1 plate. At the same time, the relative error between the numerical and experimental values of the critical load was no more than 9%.
- The post-critical work paths for the real plate and the FEM model show similar stability. This confirms the validity of the plate model developed in the numerical analysis, which made it possible to realize the issue of non-linear stability. For all analyzed plates, a change in the stiffness of the post-critical characteristic was observed with an increase in the size of the hole. The maximum decrease in the stiffness of the post-critical characteristic equal to 14% was obtained for the plate with the S1 layout, and the minimum equal to 10% was obtained for the plate with the layout S3.
- The study showed the effect of the layout of the fibers on the behavior of the compressed hole plates. It was observed that the S1 plate with composite layout [45|−45|90|0]s had more than three times lower strength compared to S2 [0|−45|45|90]s and S3 [0|90|0|90]s. This is evidenced by the displacement of the stiffest layer with the [0] layup to the plane of symmetry, thus weakening the S1 plate.
- The results of the critical and post-critical state analysis show the assumed trend. Numerical analysis was characterized by higher values compared to experimental ones. This is evidenced by the fact that the numerical analysis compared to the experiment represents ideal test conditions. At the same time, the satisfactory quantitative agreement of the results of the numerical analysis with the experimental results confirms the adequacy of the developed numerical model, which in the simulated case reproduces the behavior of the real plate with a hole.

## Figures and Tables

**Table 1:** Laminate layout [51].

Sample	Laminate Layout
S1	[45|−45|90|0|0|90|−45|45]_T_
S2	[0|−45|45|90|90|45|−45|0]_T_
S3	[0|90|0|90|90|0|90|0]_T_

**Table 2:** CFRP composite properties.

Tensile Strength	F_TU_	0°	1867 MPa
		90°	26 MPa
Tensile Modulus	E_1_	0°	131.71 GPa
	E_2_	90°	6.36 GPa
Poisson’s Ratio	ν_12_	0°	0.32
Shear Strength	F_SU_	±45°	100.15 GPa
Shear Modulus	G_12_	±45°	4.18 GPa
Compression Strength	F_CU_	0°	1531 MPa
		90°	214 MPa

## Data Availability

Data are contained within the article.
